# Supplementary material for: Mind to move: Differences in running biomechanics between sensing and intuition shod runners
Source: PLoS One. 2024 Apr 3;19(4):e0300108. doi: 10.1371/journal.pone.0300108 (PMC10990178; doi:10.1371/journal.pone.0300108)
Supplement: S1 File — (PDF) [file pone.0300108.s001.pdf]

PERSONAL PROTECTION COMMITTEE

ESTI

MEDICAL SCHOOL
Box 87900
21079 DIJON CEDEX

EXTRACT FROM THE MINUTES

THURSDAY MARCH 20, 2014 AT 5 P.M.
BOARD ROOM - CHU DIJON

ATTENDEES :

Doctors or persons qualified in biomedical research: (Category I): Dr QUENOT (Titular), Pr BONITHON-KOPP (Substitute)
General practitioners (Category II): Dr TRINH (Substitute)
Hospital Pharmacists (Category III): M AMOUREUX (Registered)
Nurses (Category IV): Ms. POUREZ (Substitute),
Qualified person due to their competence with regard to ethical issues (Category V) Ms. HUICHARD (Registered),
Psychologist (Category VI): Ms. HIDALGO (Titular)
Social worker (Category VII): Ms. VERMOT (Regular)
Persons qualified by virtue of their competence in legal matters (Category VIII): Mr. MAZEN (Substitute)
Representatives of approved associations of patients and users of the health system (Category IX): Mr LECOMTE (Registered), Ms PLASSARD (Registered)

During this meeting the file was studied:

2014/17-RCB ID: 2014-A00336-41.
<<Individualization of running training: validation of motor preferences (bioenergetic, biomechanical aspects and effects on responses to training)>> Promoter: Sarl VOLODALEN
Investigator-Coordinator: Dr Jean-Denis ROUILLON

Documents reviewed by the Committee:

Synopsis of the study 'PREFERENCES' - version 2014-1 of February 23, 2014
Information note and informed consent collection form Study 1' version 1 of 02/23/2014 Information note and informed consent collection form 'Study 2' version 1 of 02/23/2014 Study protocol 'PREFERENCES' – version 1 of February 23, 2014 Serious Adverse Event declaration form

Opinion of the Committee: FAVORABLE.

Done in Dijon, March 24, 2014

Drivet

President
Dr Jean-Pierre QUENOT
