# Supplementary material for: Mind to move: Differences in running biomechanics between sensing and intuition shod runners
Source: PLoS One. 2024 Apr 3;19(4):e0300108. doi: 10.1371/journal.pone.0300108 (PMC10990178; doi:10.1371/journal.pone.0300108)
Supplement: Attachment — Submitted filename: review.docx [file pone.0300108.s002.docx]

**Subject:** Response to the editor and reviewer

**Manuscript title:** We run the way we are: differences in running biomechanics between Sensing and Intuition shod runners

We would like to thank the reviewers for the useful comments. The feedback provided was helpful in improving the quality of our manuscript during the revision process. We have adapted our manuscript as suggested based on the reviewers’ comments. We have provided an answer to each comment.

In addition, to facilitate the review process, we have indicated all modifications in the manuscript in RED font color, except for where we removed text. We hope that the reviewers find our responses meet their expectations.

**Reviewer 1**

Although the writing of the article is almost good, but the writing of the results has been done poorly. it is suggested

1 - The results of the normality of the data should be reported in the text in the form of a table

**Response:** Data normality and homogeneity of variance were added to the revised version of the manuscript in the form of two tables, as suggested by the reviewer.

**Table 1.** Participant characteristics (age, height, body mass, and weekly running hours) for the four Myers-Briggs Type Indicator (MBTI) axes together with their Cohen’s *d* effect size. Significant differences (*p* ≤ 0.05) are indicated in bold. Participant characteristics along each MBTI axis were compared using ANOVA and non-parametric ANOVA when data normality was not verified. Data normality and homogeneity of variance were evaluated using Kolmogorov-Smirnov and Levene's tests, respectively.

| MBTI axis | | Group | Number of participants | Age  (y) | Height  (cm) | Body mass  (kg) | Weekly running hours (h/week) |
| --- | --- | --- | --- | --- | --- | --- | --- |
| Extraversion-introversion | | Extraversion | 37 (46%) | 30 ± 12 | 176 ± 8 | 70 ± 9 | 6 ± 4 |
|  |  | Introversion | 43 (54%) | 29 ± 10 | 177 ± 7 | 71 ± 10 | 8 ± 5 |
|  |  |  | *d* | -0.06 | 0.06 | 0.12 | 0.40 |
| Sensing-intuition | | Sensing | 47 (59%) | 30 ± 12 | 177 ± 8 | 70 ± 10 | 6 ± 5 |
|  |  | Intuition | 33 (41%) | 28 ± 10 | 176 ± 7 | 70 ± 9 | 7 ± 5 |
|  |  |  | *d* | -0.15 | -0.08 | -0.02 | 0.11 |
| Thinking-feeling | | Thinking | 35 (44%) | 29 ± 11 | 179 ± 7 | 72 ± 9 | 7 ± 6 |
|  |  | Feeling | 45 (56%) | 29 ± 11 | 174 ± 8 | 68 ± 9 | 6 ± 4 |
|  |  |  | *d* | -0.01 | -0.66 | -0.43 | -0.27 |
| Judging-perceiving | | Judging | 41 (51%) | 29 ± 12 | 177 ± 8 | 70 ± 10 | 7 ± 5 |
|  |  | Perceiving | 39 (49%) | 30 ± 10 | 175 ± 8 | 70 ± 9 | 6 ± 5 |
|  |  |  | *d* | 0.07 | -0.24 | 0.06 | -0.11 |
| *p*-values | ANOVA Main MBTI effect | | | 0.18 | 0.07 | 0.46 | 0.16 |
|  | Normality test | | | **<0.001** | 0.98 | 0.23 | **0.04** |
|  | Homogeneity test | | | 0.31 | 0.82 | 0.82 | 0.74 |

Note. Data are presented as mean ± standard deviation.

**Table 2.** Data normality and homogeneity of variance (*p*-values) of the biomechanical variables evaluated using Kolmogorov-Smirnov and Levene's tests, respectively. No significant difference was reported (*p* > 0.05).

| Biomechanical variable | Test (*p*) | Running speed (m/s) | | |
| --- | --- | --- | --- | --- |
|  |  | 3.3 | 4.2 | 5.0 |
| $t_{c}$ | Normality | 0.73 | 0.97 | 0.90 |
|  | Homogeneity | 0.70 | 0.50 | 0.29 |
| $t_{f}$ | Normality | 0.63 | 0.98 | 0.93 |
|  | Homogeneity | 0.66 | 0.42 | 0.35 |
| DF | Normality | 0.86 | 0.85 | 0.81 |
|  | Homogeneity | 0.46 | 0.17 | 0.33 |
| SF | Normality | 0.64 | 0.98 | 0.51 |
|  | Homogeneity | 0.44 | 0.15 | 0.19 |
| $k_{\mathrm{leg}}$ | Normality | 0.61 | 0.83 | 0.82 |
|  | Homogeneity | 0.57 | 0.46 | 0.28 |
| $F_{\max}$ | Normality | 0.64 | 0.22 | 0.45 |
|  | Homogeneity | 0.80 | 0.54 | 0.79 |
| $\Delta L$ | Normality | 0.44 | 0.31 | 0.07 |
|  | Homogeneity | 0.18 | 0.13 | 0.07 |

Note. contact time: $t_{c}$, flight time: $t_{f}$, duty factor: DF, step frequency: SF, leg stiffness $k_{\mathrm{leg}}$, maximal vertical ground reaction force: $F_{\max}$, and maximal leg compression of the spring during stance: $\Delta L$.

2 - The demographic characteristics of the participants should be mentioned and reported in the table

**Response:** As suggested by the reviewer, a table including demographic characteristics was added to the revised version of the manuscript. Moreover, we also indicated that runners were “white” Caucasians in the paragraph Participants.

Line 140

*All runners identified as “white” Caucasians.*

**Table 1.** Participant characteristics (age, height, body mass, and weekly running hours) for the four Myers-Briggs Type Indicator (MBTI) axes together with their Cohen’s *d* effect size. Significant differences (*p* ≤ 0.05) are indicated in bold. Participant characteristics along each MBTI axis were compared using ANOVA and non-parametric ANOVA when data normality was not verified. Data normality and homogeneity of variance were evaluated using Kolmogorov-Smirnov and Levene's tests, respectively.

| MBTI axis | | Group | Number of participants | Age  (y) | Height  (cm) | Body mass  (kg) | Weekly running hours (h/week) |
| --- | --- | --- | --- | --- | --- | --- | --- |
| Extraversion-introversion | | Extraversion | 37 (46%) | 30 ± 12 | 176 ± 8 | 70 ± 9 | 6 ± 4 |
|  |  | Introversion | 43 (54%) | 29 ± 10 | 177 ± 7 | 71 ± 10 | 8 ± 5 |
|  |  |  | *d* | -0.06 | 0.06 | 0.12 | 0.40 |
| Sensing-intuition | | Sensing | 47 (59%) | 30 ± 12 | 177 ± 8 | 70 ± 10 | 6 ± 5 |
|  |  | Intuition | 33 (41%) | 28 ± 10 | 176 ± 7 | 70 ± 9 | 7 ± 5 |
|  |  |  | *d* | -0.15 | -0.08 | -0.02 | 0.11 |
| Thinking-feeling | | Thinking | 35 (44%) | 29 ± 11 | 179 ± 7 | 72 ± 9 | 7 ± 6 |
|  |  | Feeling | 45 (56%) | 29 ± 11 | 174 ± 8 | 68 ± 9 | 6 ± 4 |
|  |  |  | *d* | -0.01 | -0.66 | -0.43 | -0.27 |
| Judging-perceiving | | Judging | 41 (51%) | 29 ± 12 | 177 ± 8 | 70 ± 10 | 7 ± 5 |
|  |  | Perceiving | 39 (49%) | 30 ± 10 | 175 ± 8 | 70 ± 9 | 6 ± 5 |
|  |  |  | *d* | 0.07 | -0.24 | 0.06 | -0.11 |
| *p*-values | ANOVA Main MBTI effect | | | 0.18 | 0.07 | 0.46 | 0.16 |
|  | Normality test | | | **<0.001** | 0.98 | 0.23 | **0.04** |
|  | Homogeneity test | | | 0.31 | 0.82 | 0.82 | 0.74 |

Note. Data are presented as mean ± standard deviation.

3 - The entry and exit criteria of people participating in the study should be given.

**Response:** Inclusion and exclusion criteria were clarified in the revised version of the manuscript.

Lines 140-143

*For study inclusion, participants were required to run at least one hour per week and to be in good self-reported general health with no current or recent (<6 months) musculoskeletal injuries. However, nothing specific about their spontaneous running pattern such as their foot-strike pattern was required.*

4 - I suggest putting a diagram of how to assessment running speed in the text.

**Response:** We thank the reviewer for this comment. However, we do believe that such diagram would not add enough valuable information in the present manuscript because the experimental design was quite simple. Nonetheless, the description of the experimental design was modified to improve its clarity.

Lines 156-163

*Subsequently, participants completed three randomized 50-m running-trials at speeds of 3.3, 4.2, and 5 m·s^−1^ starting from a standing-still position (2-min rest period between trials). These running speeds were chosen because they represent low, moderate, and high endurance running speeds. Speed was monitored using photoelectric cells (Racetime2, MicroGate, Timing and Sport, Bolzano, Italy) placed at the 20 and 40-m marks. A running trial was accepted if the monitored speed was within ± 5% of the requested speed and repeated otherwise after a 2-min rest period. Less than 15% of the trials were discarded.*

In general, the two parts of the study method and the results need a general review.

**Response:** As correctly pointed out by the reviewer, the materials and methods and results sections were entirely reviewed in the revised version of the manuscript**.**

Lines 134-297

***Materials and Methods***

***Participants***

*Eighty recreational endurance runners with regular running training, 67 males (age: 29.3 ± 11.1 years, height: 178.2 ± 6.4 cm, body mass: 72.0 ± 8.5 kg, and weekly running hours: 6.4 ± 3.8 h·week^−1^) and 13 females (age: 29.8 ± 11.6 years, height: 167.2 ± 6.9 cm, body mass: 60.8 ± 9.1 kg, and weekly running hours: 8.5 ± 7.8 h·week^−1^), participated in this study. All runners identified as “white” Caucasians. For study inclusion, participants were required to run at least one hour per week and to be in good self-reported general health with no current or recent (<6 months) musculoskeletal injuries. However, nothing specific about their spontaneous running pattern such as their foot-strike pattern was required. All participants completed the study on a voluntary basis but were not informed of the study’s specific purpose. The university’s institutional review board (Comité de Protection des Personnes Est 1 (CPP EST 1) approved the protocol prior to participant recruitment (ID RCB 2014-A00336-41), and the study was conducted in accordance with the latest amendments of the Declaration of Helsinki. Participants were recruited between the September 1^st^ and November 30^th^ of 2014. Each participant underwent two experimental sessions within one week: a running biomechanical analysis during the first session, and a personality traits assessment during the second one. All participants wore their habitual running shoes during the biomechanical analysis.*

***Assessment of biomechanical variables***

*After providing written informed consent, participants performed a 10-min warm-up run at a self-selected speed (range: 2.5−3.5 m·s^−1^) on an indoor athletic track. Subsequently, participants completed three randomized 50-m running-trials at speeds of 3.3, 4.2, and 5 m·s^−1^ starting from a standing-still position (2-min rest period between trials). These running speeds were chosen because they represent low, moderate, and high endurance running speeds. Speed was monitored using photoelectric cells (Racetime2, MicroGate, Timing and Sport, Bolzano, Italy) placed at the 20 and 40-m marks. A running trial was accepted if the monitored speed was within ± 5% of the requested speed and repeated otherwise after a 2-min rest period. Less than 15% of the trials were discarded. The Optojump^®^ photoelectric cells (MicroGate Timing and Sport, Bolzano, Italy) were used to measure t_c_ (in ms) and t_f_ (in ms) between the 20 and 40-m marks. The cells consist of two parallel bars which were set 1 m apart and were connected to a personal computer. One bar acts as a transmitter unit containing light emitting diodes positioned 3 mm above the ground, whereas the other bar acts as the receiver unit. When the light is interrupted by an individual’s foot during running, a timer within the Optojump system records time with a precision of 1 ms (sampling frequency of 1000 Hz). This allows measuring t_c_ as the time that the light is interrupted and t_f_ as the time between interruptions. As for each participant, the average value over the 20-m distance was computed for t_c_ and t_f_ and used in what follows. The test-retest reliability of the Optojump system was demonstrated to be excellent, with low coefficients of variation (2.7%) and high intraclass correlation coefficients (range: 0.982 to 0.989) (20).*

*Then, swing time (t_s_, in ms), corresponding to the time during which a foot is not in contact with the ground within a running stride, was calculated as two consecutives t_f_ and one t_c_, i.e.,* $t_{s}={2t}_{f}+t_{c}$*. SF was calculated as follows:*

$$SF=\frac{1}{t_{c} + t_{f}},$$

*and DF, reflecting the relative contribution of t_c_ during the running stride, as (21, 22):*

$$DF=\frac{t_{c}}{(t_{c}+t_{s})}.$$

*Furthermore, the spring-mass characteristics of the lower limb were estimated using a sine-wave model as defined by Morin, Dalleau (23). More explicitly, leg stiffness (k_leg_) was calculated as:*

$$k_{leg}=\frac{F_{max}}{\Delta L}.$$

$F_{max}$ *represents the maximal vertical ground reaction force and was estimated using* $F_{max}=mg\frac{\pi}{2}\left( \frac{t_{f}}{t_{c}}+1 \right)$*, where m is body mass and g is gravitational acceleration.* $\Delta L$ *represents the maximal leg compression of the spring during stance and was modeled as:*

$$\Delta L=L-\sqrt{L^{2}-\left( \frac{st_{c}}{2} \right)^{2}}+\Delta z,$$

*where* $\Delta z=\frac{F_{max}}{m}\frac{t_{c}^{2}}{\pi^{2}}-g\frac{t_{c}^{2}}{8}$ *is the absolute displacement of the center of mass during stance,* $s$ *the running speed, and* $L$ *the participant’s leg length, estimated as 0.53 of body height.*

***Assessment of personality traits***

*Based on the answers to 93 questions, the MBTI classifies personality traits into one of two possible categories along four axes: extraversion-introversion (favorite world); sensing-intuition (information processing preference); thinking-feeling (decision making); and judging-perceiving (structure). Together, these axes influence how an individual perceives a situation and decides on a course of action. The MBTI has demonstrated excellent stability with test-retest correlations between 0.83 and 0.97 over a 4-week interval, exceeding the stability of many established trait measures, and between 0.77 and 0.84 over a 9-month interval (24). Moreover, each dichotomy showed an agreement of 84 to 96% over 4 weeks, with a median agreement of 90% (17). Given potential context-dependent results of the MBTI (24), the personality traits of participants were reassessed through a face-to-face meeting lasting approximately one hour, conducted by an MBTI-certified practitioner, to ensure data quality.*

*MBTI correlates well with the NEO Personality Inventory, another widely used personality assessment tool that examines the Big Five personality traits (25, 26). MBTI has been utilized to assess personality traits in diverse context, including a study involving middle-age male runners (13) and applications in sport psychology assessment, particularly for enhancing team cohesion and understanding team dynamics (27). More recently, MBTI has been employed to describe the personality profiles for talent identification in elementary and junior high school students (28) and students in the field of recreation and leisure within the general population (29).*

***Statistics***

*Sample size calculations determined that 80 participants were required for this study, assuming moderate effect sizes (~0.5) for biomechanical differences between MBTI axes, an α error of 0.05, and a power of 0.8 (30). Descriptive statistics are presented as mean ± standard deviation. Data normality and homogeneity of variance were evaluated using Kolmogorov-Smirnov and Levene's tests, respectively. Participant characteristics were compared along each MBTI axis using ANOVA and non-parametric ANOVA when data normality was not verified. Repeated-measures ANOVA (speed x MBTI axes) with Mauchly’s correction for sphericity and employing Holm corrections for pair-wise post-hoc comparisons were used to investigate the effect of each MBTI axis on the biomechanical variables (*$t_{c}$*,* $t_{f}$*, DF, SF,* $k_{leg}$*,* $F_{max}$*, and* $\Delta L$*) while accounting for the effect of running speed. 95% confidence intervals [lower, upper] of mean differences (∆s) were calculated for each significant post-hoc comparison along the MBTI axes. Cohen's d effect sizes were calculated for participant characteristics along the four MBTI axes and for each significant post-hoc comparison. Effect sizes were classified as small, moderate, or large based on the magnitude of d values (0.2, 0.5, and 0.8, respectively) (31). Statistical analysis was conducted using Jamovi (v1.6.23, available at https://www.jamovi.org), with significance set at α ≤ 0.05.*

***Results***

*Classifications of participants along the four MBTI axes are reported in Table 1. Normality and homogeneity of variance were verified for age, height, body mass, and weekly running hours (p ≥ 0.07; Table 1) except for age and weekly running hours which were not normally distributed (p ≤ 0.04; Table 1). ANOVA and non-parametric ANOVA results indicated no main effect of the MBTI axes on age, height, body mass, and weekly running hours (p ≥ 0.07; Table 1), suggesting that these characteristics were similar across each MBTI axis. Effect sizes were small for age, height, body mass, and weekly running hours between the categories of each MBTI axis (|d| ≤ 0.27; Table 1), except for weekly running hours of the extraversion-introversion axis, and height and body mass of the thinking-feeling axis which were moderate (0.40 ≤ |d| ≤ 0.66; Table 1).*

**Table 1.** Participant characteristics (age, height, body mass, and weekly running hours) for the four Myers-Briggs Type Indicator (MBTI) axes together with their Cohen’s *d* effect size. Significant differences (*p* ≤ 0.05) are indicated in bold. Participant characteristics along each MBTI axis were compared using ANOVA and non-parametric ANOVA when data normality was not verified. Data normality and homogeneity of variance were evaluated using Kolmogorov-Smirnov and Levene's tests, respectively.

| MBTI axis | | Group | Number of participants | Age  (y) | Height  (cm) | Body mass  (kg) | Weekly running hours (h/week) |
| --- | --- | --- | --- | --- | --- | --- | --- |
| Extraversion-introversion | | Extraversion | 37 (46%) | 30 ± 12 | 176 ± 8 | 70 ± 9 | 6 ± 4 |
|  |  | Introversion | 43 (54%) | 29 ± 10 | 177 ± 7 | 71 ± 10 | 8 ± 5 |
|  |  |  | *d* | -0.06 | 0.06 | 0.12 | 0.40 |
| Sensing-intuition | | Sensing | 47 (59%) | 30 ± 12 | 177 ± 8 | 70 ± 10 | 6 ± 5 |
|  |  | Intuition | 33 (41%) | 28 ± 10 | 176 ± 7 | 70 ± 9 | 7 ± 5 |
|  |  |  | *d* | -0.15 | -0.08 | -0.02 | 0.11 |
| Thinking-feeling | | Thinking | 35 (44%) | 29 ± 11 | 179 ± 7 | 72 ± 9 | 7 ± 6 |
|  |  | Feeling | 45 (56%) | 29 ± 11 | 174 ± 8 | 68 ± 9 | 6 ± 4 |
|  |  |  | *d* | -0.01 | -0.66 | -0.43 | -0.27 |
| Judging-perceiving | | Judging | 41 (51%) | 29 ± 12 | 177 ± 8 | 70 ± 10 | 7 ± 5 |
|  |  | Perceiving | 39 (49%) | 30 ± 10 | 175 ± 8 | 70 ± 9 | 6 ± 5 |
|  |  |  | *d* | 0.07 | -0.24 | 0.06 | -0.11 |
| *p*-values | ANOVA Main MBTI effect | | | 0.18 | 0.07 | 0.46 | 0.16 |
|  | Normality test | | | **<0.001** | 0.98 | 0.23 | **0.04** |
|  | Homogeneity test | | | 0.31 | 0.82 | 0.82 | 0.74 |

Note. Data are presented as mean ± standard deviation.

*Data normality and homogeneity of variance of the biomechanical variables were all verified (p ≥ 0.07; Table 2).*

**Table 2.** Data normality and homogeneity of variance (*p*-values) of the biomechanical variables evaluated using Kolmogorov-Smirnov and Levene's tests, respectively. No significant difference was reported (*p* > 0.05).

| Biomechanical variable | Test (*p*) | Running speed (m/s) | | |
| --- | --- | --- | --- | --- |
|  |  | 3.3 | 4.2 | 5.0 |
| $t_{c}$ | Normality | 0.73 | 0.97 | 0.90 |
|  | Homogeneity | 0.70 | 0.50 | 0.29 |
| $t_{f}$ | Normality | 0.63 | 0.98 | 0.93 |
|  | Homogeneity | 0.66 | 0.42 | 0.35 |
| DF | Normality | 0.86 | 0.85 | 0.81 |
|  | Homogeneity | 0.46 | 0.17 | 0.33 |
| SF | Normality | 0.64 | 0.98 | 0.51 |
|  | Homogeneity | 0.44 | 0.15 | 0.19 |
| $k_{\mathrm{leg}}$ | Normality | 0.61 | 0.83 | 0.82 |
|  | Homogeneity | 0.57 | 0.46 | 0.28 |
| $F_{\max}$ | Normality | 0.64 | 0.22 | 0.45 |
|  | Homogeneity | 0.80 | 0.54 | 0.79 |
| $\Delta L$ | Normality | 0.44 | 0.31 | 0.07 |
|  | Homogeneity | 0.18 | 0.13 | 0.07 |

Note. contact time: $t_{c}$, flight time: $t_{f}$, duty factor: DF, step frequency: SF, leg stiffness $k_{\mathrm{leg}}$, maximal vertical ground reaction force: $F_{\max}$, and maximal leg compression of the spring during stance: $\Delta L$.

*A speed x sensing-intuition axis interaction effect was observed for* $t_{c}$ *(p = 0.02), with no other significant interaction effects reported (other interactions: p ≥ 0.06). Pair-wise post-hoc comparisons revealed significantly shorter* $t_{c}$ *for intuition runners compared to sensing runners at all running speeds examined (p ≤ 0.02; Fig. 1a) with moderate to large effect sizes (0.73 ≤ d ≤ 1.02).*

*The sensing-intuition axis reported significant differences among the biomechanical variables, leading to sensing runners showing a longer* $t_{c}$ *(∆ = 13 ms [5 ms, 21 ms]; p = 0.002; small effect size; d = 0.44), shorter* $t_{f}$ *(∆ = -16 ms [-22 ms, -10 ms]; p < 0.001; moderate effect size; d = -0.73), higher DF (∆ = 2.1% [1.3%, 2.9%]; p < 0.001; moderate effect size; d = 0.66), smaller* $k_{leg}$ *(∆ = -1.1 kN [-1.5 kN, -0.7 kN]; p = 0.01; moderate effect size; d = -0.68), and larger* $\Delta L$ *(∆ = 1.4 cm [0.7 cm, 2.1 cm]; p = 0.02; moderate effect size; d = 0.55) than intuition runners (Fig. 1 and Table 3). The other three axes did not report any significant differences among the biomechanical variables (p ≥ 0.09).*

**Figure 1. Boxplots of the temporal characteristics of the running form for intuition and sensing runners.** **a**, contact time ($t_{c}$); **b**, flight time ($t_{f}$); **c**, duty factor (DF). Intuition runners (blue boxes; left side) exhibited shorter $t_{c}$ (*p* = 0.002), longer $t_{f}$ (*p* < 0.001), and lower DF (*p* < 0.001) than sensing runners (red boxes; right side). A significant speed x sensing-intuition axis interaction effect was observed for $t_{c}$ (*p* = 0.02). * Significantly shorter $t_{c}$ for intuition than sensing runners, as reported by the pair-wise post-hoc comparisons (*p* ≤ 0.02).

**Table 3.** Step frequency (SF), leg stiffness ($k_{\mathrm{leg}}$), maximal vertical ground reaction force ($F_{\max}$), and maximal leg compression of the spring during stance ($\Delta L$) for sensing and intuition runners at three tested speeds. Significant sensing-intuition axis effects (*p* ≤ 0.05) identified by the two-way repeated measures ANOVA are indicated in bold.

| Biomechanical variable | Sensing-intuition  group | Running speed (m/s) | | | Sensing-intuition axis effect *(p)* |
| --- | --- | --- | --- | --- | --- |
|  |  | 3.3 | 4.2 | 5.0 |  |
| SF (Hz) | Sensing  Intuition | 2.7±0.1  2.7±0.1 | 2.8±0.2  2.8±0.1 | 3.0±0.2  2.9±0.1 | 0.21 |
| $k_{\mathrm{leg}}$ (kN/m) | Sensing  Intuition | 8.1±1.7  9.3±1.4 | 8.1±1.6  9.0±1.4 | 8.3±1.6  9.4±1.9 | **0.01** |
| $F_{\max}$ (N) | Sensing  Intuition | 1454±226  1554±192 | 1612±231  1699±213 | 1752±249  1860±241 | 0.09 |
| $\Delta L$ (cm) | Sensing  Intuition | 18±2  17±1 | 20±2  19±2 | 22±3  20±2 | **0.02** |

Note. data are presented as mean ± standard deviation.

*All the biomechanical variables investigated herein (*$t_{c}$*,* $t_{f}$*, DF, SF,* $k_{leg}$*,* $F_{max}$*, and* $\Delta L$*) reported a significant running speed effect (p ≤ 0.02), where* $t_{c}$ *and DF decreased with increasing speed while* $t_{f}$*, SF,* $k_{leg}$*,* $F_{max}$*, and* $\Delta L$ *increased with increasing running speed.*

**Reviewer 2**

The study aimed to explore the potential association between biomechanical variables and personality traits assessed through the Myers-Briggs Type Indicator test in a cohort of 80 runners. Although it is a very interesting study, there are several significant concerns that need to be addressed.

**Response:** We thank the reviewer for this positive general feedback. Comments have been addressed in what follows.

1. There are several areas in the Abstract section that require improvement.

1) please add some background information to better emphasize the novelty and significance of the research.

**Response:** We thank the reviewer for this comment. Background information was added to the abstract to better emphasize the novelty and significance of the research.

Lines 39-43

*Delving into the complexities of embodied cognition unveils the intertwined influence of mind, body, and environment. The connection of physical activity with cognition, mood, and thought sparks a hypothesis linking motion and personality traits. Hence, this study explored whether personality traits could be linked to biomechanical variables characterizing running forms naturally embraced by shod runners.*

2) there is no description about the research methods here, which makes it hard to understand the results of this study.

**Response:** As suggested by the reviewer, research methods were inserted to the abstract.

Lines 43-48

*To do so, 80 runners completed three randomized 50-m running-trials at 3.3, 4.2, and 5 m·s^−1^ during which their running biomechanics was evaluated. In addition, participants’ personality traits were assessed through the Myers-Briggs Type Indicator (MBTI) test. The MBTI classifies personality traits into one of two possible categories along four axes: extraversion-introversion; sensing-intuition; thinking-feeling; and judging-perceiving.*

3) The conclusion should be further strengthened based on the main findings of the study. The study conclusion should be concise and indicate the most important findings of the research;

**Response:** As suggested by the reviewer, the conclusion of the abstract has been further strengthened.

Lines 49-64

*This exploratory study offers compelling evidence that personality traits, specifically sensing and intuition, are associated with distinct running biomechanics. Individuals classified as sensing demonstrated a more grounded running style characterized by prolonged ground contact time, shorter flight time, higher duty factor, and greater maximal leg compression during stance compared to intuition individuals (p≤0.02). Conversely, intuition runners exhibited a more dynamic and springy running style with a shorter ground contact time and higher leg stiffness than their sensing counterparts (p≤0.02). Sensing individuals tend to focus on concrete facts and physical realities while intuition individuals emphasize abstract concepts and patterns of information. These results suggest that runners with sensing and intuition personality traits differ in their ability to use their lower limb structures as springs. Intuition runners appeared to rely more in the stretch-shortening cycle to energetically optimize their running style while sensing runners seemed to optimize running economy by promoting more forward progression than vertical oscillations. Although no causal or predictive conclusions could be drawn from this specific study’s design, this study underscores the intriguing interplay between an individual’s personality traits and their preferred movement patterns.*

4) it would be beneficial to improve the quality of the keywords used to make it easier for researchers and other interested parties to locate information relevant to the research topic.

**Response:** Keywords have been improved according to the suggestion of the reviewer.

Lines 66-67

***Keywords:*** *personality types; Myers-Briggs type indicator test; personality and behavior; running pattern; running style; psychology; embodied cognition*

2. In the Introduction section, the authors described their objective to explores the potential association between biomechanical variables and personality traits. However, the explanation of the research gap was insufficient, and the novelty of the study was not clearly communicated. Merely stating that no research has been conducted on this topic does not necessarily indicate the significance of the study. It is essential to explain what new insights this study can bring to the existing knowledge in this field and how it is relevant to practical situations. In addition, please add the corresponding hypotheses at the end of this session.

**Response:** We thank the reviewer for this comment. The introduction has been rewritten to emphasize the new insights this study can bring to the existing knowledge in this field and how it is relevant to practical situations. Moreover, a hypothesis was added at the end of the introduction section of the revised version of the manuscript.

Lines 69-132

*Embodied cognition, a compelling theoretical framework in cognitive science, challenges conventional notions that divorce the mind from the body (1). This paradigm asserts a symbiotic relationship between cognitive processes and the physical body, underscoring the significance of sensory and motor experiences in shaping mental functions (2). Unlike traditional views that confine cognition to the brain, embodied cognition recognizes the profound impact of the body's interactions with the environment on cognitive phenomena (3). This approach emphasizes both bottom-up processes, where sensory information informs cognitive processes, and top-down influences, where higher-order cognitive functions shape our perception and interaction with the world.*

*Exploring the intricate dynamics of embodied cognition sheds light on the reciprocal influence between the mind, body, and the vibrant world they jointly navigate. For example, an investigation revealed that the extraverted-introverted continuum influences upright posture, with 96% of extraverted individuals maintaining an “ideally aligned” posture, while 83% of introverted individuals exhibit a kyphosis-lordosis posture (4). Furthermore, higher levels of extraversion and conscientiousness have been linked to increased physical activity levels (5) and to faster walking speeds (6, 7), suggesting that the way individuals move may reflect their underlying personality traits. Conscientiousness, notably, has shown the capacity to mitigate the age-related decline in walking speed (6, 8). Research has also uncovered that personality traits manifest in an individual’s walking gait (9). For instance, it was observed that greater pelvis motion in the horizontal plane during walking is associated with greater agreeableness in females, while, for males, greater thorax motion in the horizontal plane is linked with extraversion (9). In a recent development, machine learning techniques were employed with notable accuracy to assess personality traits through the analysis of gait recorded using videos (10) or smartphone sensors (11).*

*Extending this line of inquiry into the realm of running, there are indications that personality traits may influence a runner’s likelihood of engaging in barefoot running (12). This study’s results suggest that runners who have attempted barefoot running tend to exhibit traits such as openness, imagination, unconventionality, and curiosity while displaying lower levels of conscientiousness, responsibility, dependability, and order in comparison to shod runners. Additionally, research on middle-aged male runners has identified common personality profiles and associated positive self-perception with long-term involvement in running and training (13). Runners demonstrated heightened intelligence, creativity, self-sufficiency, sobriety, and forthrightness compared to the general population, embodying traits of introversion, shyness, and a propensity for imaginative pursuits in their personality composition (14). Besides, a prospective study found that runners with high scores on the type A behaviour (characterized by agitation, hostility, rapid speech, and an extremely competitive nature) screening questionnaire experienced significantly more injuries, especially multiple injuries (15). Nonetheless, limited information exists on the personality of recreational runners, relying mostly on old studies. This underscores the imperative for contemporary, original research specifically addressing the personality traits of recreational runners (16).*

*Personality traits could be effectively classified into one of two possible categories along various axes using the Myers-Briggs Type Indicator (MBTI) test, a tool rooted in Jungʼs psychology (17). Besides, recent research suggested that the duty factor (DF), i.e., the proportion of time spent in contact with the ground during a running stride, plays a pivotal role in illustrating two distinct spontaneous running forms in recreational runners (18, 19). Low DF runners were shown to exhibit a shorter contact time (t_c_) and larger vertical oscillation of the center of mass during flight time (t_f_), favoring elastic energy reuse. Conversely, high DF runners demonstrated a longer t_c_ and reduced work against gravity to promote forward progression. Both strategies could be efficiently employed at endurance running speeds, leading to similar running economy measures (18).*

*In the present study, we delve into the intriguing realm of embodied cognition by exploring if an intricate connection could exist between personality traits and spontaneous running patterns of shod runners. Indeed, the aim of this study was to explore whether the two categories of personality traits within the various MBTI axes could be linked to biomechanical variables that characterize two distinct running forms naturally embraced by individuals. This exploration should shed light on the complex relationship between who we are and how we run. We hypothesized that personality traits would demonstrate association with spontaneous running patterns.*

3. The Methods section of the study provides limited information on the participants and data analysis. To improve the clarity and comprehensiveness of this section, the following suggestions are recommended.

1) the inclusion criteria were relatively broad, for example, how about the running strike patterns of these participants?

**Response:** There were no extra inclusion criteria for this study. This was clarified in the revised version of the manuscript.

Lines 140-143

*All runners identified as “white” Caucasians. For study inclusion, participants were required to run at least one hour per week and to be in good self-reported general health with no current or recent (<6 months) musculoskeletal injuries. However, nothing specific about their spontaneous running pattern such as their foot-strike pattern was required.*

2) “3.3, 4.2, 5 m/s”, why did the authors choose these running speeds? To represent low, moderate, and high running speeds? For what purposes?

**Response:** As correctly pointed out by the reviewer, these three running speeds were chosen to represent low, moderate, and high endurance running speeds.

Lines 158-160

*These running speeds were chosen because they represent low, moderate, and high endurance running speeds.*

3) did all participants wear the same running shoes during the test?

**Response:** Shoes were not imposed during the present study. Hence, participants wore their habitual running shoes during the biomechanical analysis. This clarification was added to the revised version of the manuscript. Nonetheless, a comment in the limitations was added to mention that standardized shoe might have led to different study outcomes.

Lines 151-152

*All participants wore their habitual running shoes during the biomechanical analysis.*

Lines 432-438

*Furthermore, participants wore their own running shoes during testing, which could be confounding our results. Given that differences in footwear characteristics can underpin differences in running biomechanics (48), using a standardized shoe might have led to different study outcomes in terms of running biomechanics. Nonetheless, recreational runners are more comfortable wearing their own shoes (49), and show individual responses to novel footwear (49, 50) and cushioning properties (51).*

4) how did the optical measurement system capture the motion and calculate the corresponding parameters?

**Response:** More information about the Optojump recording system was added to the revised version of the manuscript, according to the suggestion of the reviewer.

Lines 163-178

*The Optojump^®^ photoelectric cells (MicroGate Timing and Sport, Bolzano, Italy) were used to measure t_c_ (in ms) and t_f_ (in ms) between the 20 and 40-m marks. The cells consist of two parallel bars which were set 1 m apart and were connected to a personal computer. One bar acts as a transmitter unit containing light emitting diodes positioned 3 mm above the ground, whereas the other bar acts as the receiver unit. When the light is interrupted by an individual’s foot during running, a timer within the Optojump system records time with a precision of 1 ms (sampling frequency of 1000 Hz). This allows measuring t_c_ as the time that the light is interrupted and t_f_ as the time between interruptions. Swing time (t_s_, in ms), corresponding to the time during which a foot is not in contact with the ground within a running stride, was calculated as two consecutives t_f_  and one t_c_, i.e.,* $t_{s}={2t}_{f}+t_{c}$*.*

5) “a 20-m distance”, I don’t think that 20-m distance is enough to reach a stable 5m/s running speed.

**Response:** We thank the reviewer for this comment. Photoelectric cells monitoring speed were positioned at the 20 and 40-m marks, similarly to the Optojump cells which measured running biomechanics. Running trials were accepted only if the speed monitored within the 20-m distance was within 5% of the requested speed. Hence, the average of the biomechanical variables for the 20-m distance represent the monitored speed.

To further make sure each participant indirectly had a stable speed, we calculated the average value over participants for the coefficient of variations (standard deviation divided by mean value) of flight, contact, and step times within the 20-m distance. These coefficients represent the variability of the running stride within individuals. The coefficient of variations for flight, contact, and step times were 8%, 4%, and 2% at 3.3 m/s, 6%, 4%, and 3% at 4.2 m/s, and 6%, 3%, and 3% at 5 m/s. These numbers were much smaller than the coefficient of variations representing the variability of the running stride between individuals (flight, contact, and step times: 20%, 14%, and 13% at 3.3 m/s, 8%, 7%, and 7% at 4.2 m/s, and 5%, 5%, and 5% at 5 m/s). Hence, we can conclude that the 20-m distance was long enough to reach stable running speeds of 3.3, 4.2, and 5 m/s.

6) this study explores the potential association between biomechanical variables and personality traits, thus how did the authors avoid the influence of other conditions? for example, the mood of the participants may have some influence on the gait features.

**Response:** We thank the reviewer for this comment. We agree with the reviewer that emotion, mood, or facial expression could have influenced the running biomechanics of participants. However, we found no scientific evidence that these factors could impact the gait features. Nonetheless, this was added in the limitation section of the revised version of the manuscript.

Lines 418-425

*Next, several factors, such as emotion, mood, or facial expression, which were not measured herein, might have partly confound the results of the present study. For instance, Williams, Exell (48) reported that sadness might increase running asymmetry while anger might facilitate symmetry and Brick, McElhinney (49) showed that oxygen consumption was lower when smiling than frowning during running and perceived effort was higher when frowning than smiling. However, to the best of authors knowledge, there was no direct scientific evidence that these factors could influence the biomechanical variables measured herein.*

4. For the results of this study,

1) it is suggested that a table could be used to clearly present the basic information of the participants and the results of the personality traits assessment.

**Response:** As suggested by the reviewer, a table including participant characteristics and results of the personality traits assessment was added to the revised version of the manuscript.

**Table 1.** Participant characteristics (age, height, body mass, and weekly running hours) for the four Myers-Briggs Type Indicator (MBTI) axes together with their Cohen’s *d* effect size. Significant differences (*p* ≤ 0.05) are indicated in bold. Participant characteristics along each MBTI axis were compared using ANOVA and non-parametric ANOVA when data normality was not verified. Data normality and homogeneity of variance were evaluated using Kolmogorov-Smirnov and Levene's tests, respectively.

| MBTI axis | | Group | Number of participants | Age  (y) | Height  (cm) | Body mass  (kg) | Weekly running hours (h/week) |
| --- | --- | --- | --- | --- | --- | --- | --- |
| Extraversion-introversion | | Extraversion | 37 (46%) | 30 ± 12 | 176 ± 8 | 70 ± 9 | 6 ± 4 |
|  |  | Introversion | 43 (54%) | 29 ± 10 | 177 ± 7 | 71 ± 10 | 8 ± 5 |
|  |  |  | *d* | -0.06 | 0.06 | 0.12 | 0.40 |
| Sensing-intuition | | Sensing | 47 (59%) | 30 ± 12 | 177 ± 8 | 70 ± 10 | 6 ± 5 |
|  |  | Intuition | 33 (41%) | 28 ± 10 | 176 ± 7 | 70 ± 9 | 7 ± 5 |
|  |  |  | *d* | -0.15 | -0.08 | -0.02 | 0.11 |
| Thinking-feeling | | Thinking | 35 (44%) | 29 ± 11 | 179 ± 7 | 72 ± 9 | 7 ± 6 |
|  |  | Feeling | 45 (56%) | 29 ± 11 | 174 ± 8 | 68 ± 9 | 6 ± 4 |
|  |  |  | *d* | -0.01 | -0.66 | -0.43 | -0.27 |
| Judging-perceiving | | Judging | 41 (51%) | 29 ± 12 | 177 ± 8 | 70 ± 10 | 7 ± 5 |
|  |  | Perceiving | 39 (49%) | 30 ± 10 | 175 ± 8 | 70 ± 9 | 6 ± 5 |
|  |  |  | *d* | 0.07 | -0.24 | 0.06 | -0.11 |
| *p*-values | ANOVA Main MBTI effect | | | 0.18 | 0.07 | 0.46 | 0.16 |
|  | Normality test | | | **<0.001** | 0.98 | 0.23 | **0.04** |
|  | Homogeneity test | | | 0.31 | 0.82 | 0.82 | 0.74 |

Note. Data are presented as mean ± standard deviation.

2) it is suggested to further calculate the effect size of these results.

**Response:** The effect sizes of the participant characteristics were added to table summarizing the participant characteristics along each MBTI axis, as suggested by the reviewer.

**Table 1.** Participant characteristics (age, height, body mass, and weekly running hours) for the four Myers-Briggs Type Indicator (MBTI) axes together with their Cohen’s *d* effect size. Significant differences (*p* ≤ 0.05) are indicated in bold. Participant characteristics along each MBTI axis were compared using ANOVA and non-parametric ANOVA when data normality was not verified. Data normality and homogeneity of variance were evaluated using Kolmogorov-Smirnov and Levene's tests, respectively.

| MBTI axis | | Group | Number of participants | Age  (y) | Height  (cm) | Body mass  (kg) | Weekly running hours (h/week) |
| --- | --- | --- | --- | --- | --- | --- | --- |
| Extraversion-introversion | | Extraversion | 37 (46%) | 30 ± 12 | 176 ± 8 | 70 ± 9 | 6 ± 4 |
|  |  | Introversion | 43 (54%) | 29 ± 10 | 177 ± 7 | 71 ± 10 | 8 ± 5 |
|  |  |  | *d* | -0.06 | 0.06 | 0.12 | 0.40 |
| Sensing-intuition | | Sensing | 47 (59%) | 30 ± 12 | 177 ± 8 | 70 ± 10 | 6 ± 5 |
|  |  | Intuition | 33 (41%) | 28 ± 10 | 176 ± 7 | 70 ± 9 | 7 ± 5 |
|  |  |  | *d* | -0.15 | -0.08 | -0.02 | 0.11 |
| Thinking-feeling | | Thinking | 35 (44%) | 29 ± 11 | 179 ± 7 | 72 ± 9 | 7 ± 6 |
|  |  | Feeling | 45 (56%) | 29 ± 11 | 174 ± 8 | 68 ± 9 | 6 ± 4 |
|  |  |  | *d* | -0.01 | -0.66 | -0.43 | -0.27 |
| Judging-perceiving | | Judging | 41 (51%) | 29 ± 12 | 177 ± 8 | 70 ± 10 | 7 ± 5 |
|  |  | Perceiving | 39 (49%) | 30 ± 10 | 175 ± 8 | 70 ± 9 | 6 ± 5 |
|  |  |  | *d* | 0.07 | -0.24 | 0.06 | -0.11 |
| *p*-values | ANOVA Main MBTI effect | | | 0.18 | 0.07 | 0.46 | 0.16 |
|  | Normality test | | | **<0.001** | 0.98 | 0.23 | **0.04** |
|  | Homogeneity test | | | 0.31 | 0.82 | 0.82 | 0.74 |

Note. Data are presented as mean ± standard deviation.

5. Discussion, it would be interesting for future study to further investigate the gender influence during this test.

**Response:** We thank the reviewer for this comment. We would like to mention to the reviewer that the gender influence was not considered because only 13 women were involved in this study out of the 80 participants. Nonetheless, this was added in the limitation section of the revised version of the manuscript because subtle differences in personality types between males and females were observed.

Lines 425-432

*Moreover, the present study did not account for sex distinctions. Despite utilizing a relatively large sample size (n = 80), the decision was made not to differentiate between men (n = 67) and women (n = 13) to maintain simplicity and ensure an easily comprehensible manuscript, additionally given that separating the genders would have compromized statistical power. Nevertheless, future investigations should prioritize exploring the influence of sex when analyzing the connection between personality traits and running patterns, considering the demonstrated but subtle differences in personality types between males and females (48).*

6. The conclusion should be further strengthened based on the main findings of the study.

**Response:** As suggested by the reviewer, the conclusion was strengthened based on the main findings of the study.

Lines 441-455

*This exploratory study offers compelling evidence that personality traits, specifically sensing and intuition, are associated with distinct running biomechanics. Sensing runners, who pay attention to physical realities and prefer practical and specific facts, tend to adopt a more grounded running form associated with longer* $t_{c}$*, shorter* $t_{f}$*, higher DF, and larger* $\Delta L$ *than intuition runners. On the contrary, intuition runners, who prefer abstract concepts and theories, and make unconscious connections across their disciplines of knowledge, tend to opt for a more dynamic and springy running form with shorter* $t_{c}$ *and larger* $k_{leg}$ *than sensing runners. These findings implies that runners with sensing and intuition personality traits exhibit variations in their utilization of lower limb structures as springs. Intuition runners appeared to rely more on the stretch-shortening cycle, effectively optimizing their running economy through the reutilization of elastic energy, whereas sensing runners seemed to prioritize forward progression over vertical oscillations to optimize running economy. While this specific study did not permit drawing causal or predictive conclusions, it highlights the fascinating interaction between an individual's personality traits and their preferred movement patterns.*

**Reviewer 3**

General comments

In the study titled "We Run the Way We Are: Exploring the Connection Between Personality Traits and Running Biomechanics," 80 runners were included, and the relationship between biomechanical variables and personality traits, using the Myers-Briggs Type Indicator, was determined. Runners were classified into two types: "Sensing" and "Intuition." This study is intriguing as it investigates the association between biomechanics and psychological variables, offering potential insights into human running. However, the manuscript lacks a clear rationale for the study throughout. Several significant improvements are needed to enhance the study, and it is advisable to have a native speaker edit the manuscript.

**Response:** We thank the reviewer for this positive general feedback. Comments have been addressed in what follows.

Specific comments (**line numbers are based on the PDF copy)

Title
I suggest revising the title to "Differences in Running Biomechanics between Sensing and Intuition Shod Runners."

**Response:** The title was revised according to the suggestion of the reviewer. Nonetheless, the first part “We run the way we are” was kept to make it more attractive.

Lines 1-2

*We run the way we are: differences in running biomechanics between Sensing and Intuition shod runners*

Introduction
1. This section needs strengthening to provide a compelling reason for conducting the study. The authors should emphasize the significance of the current study and how the findings can improve running performance or prevent running injuries.

**Response:** The introduction has been rewritten to provide a compelling reason for conducting the study, as suggestion by the reviewer. Moreover, a paragraph about public health implications was added in the discussion.

Lines 69-132

*Embodied cognition, a compelling theoretical framework in cognitive science, challenges conventional notions that divorce the mind from the body (1). This paradigm asserts a symbiotic relationship between cognitive processes and the physical body, underscoring the significance of sensory and motor experiences in shaping mental functions (2). Unlike traditional views that confine cognition to the brain, embodied cognition recognizes the profound impact of the body's interactions with the environment on cognitive phenomena (3). This approach emphasizes both bottom-up processes, where sensory information informs cognitive processes, and top-down influences, where higher-order cognitive functions shape our perception and interaction with the world.*

*Exploring the intricate dynamics of embodied cognition sheds light on the reciprocal influence between the mind, body, and the vibrant world they jointly navigate. For example, an investigation revealed that the extraverted-introverted continuum influences upright posture, with 96% of extraverted individuals maintaining an “ideally aligned” posture, while 83% of introverted individuals exhibit a kyphosis-lordosis posture (4). Furthermore, higher levels of extraversion and conscientiousness have been linked to increased physical activity levels (5) and to faster walking speeds (6, 7), suggesting that the way individuals move may reflect their underlying personality traits. Conscientiousness, notably, has shown the capacity to mitigate the age-related decline in walking speed (6, 8). Research has also uncovered that personality traits manifest in an individual’s walking gait (9). For instance, it was observed that greater pelvis motion in the horizontal plane during walking is associated with greater agreeableness in females, while, for males, greater thorax motion in the horizontal plane is linked with extraversion (9). In a recent development, machine learning techniques were employed with notable accuracy to assess personality traits through the analysis of gait recorded using videos (10) or smartphone sensors (11).*

*Extending this line of inquiry into the realm of running, there are indications that personality traits may influence a runner’s likelihood of engaging in barefoot running (12). This study’s results suggest that runners who have attempted barefoot running tend to exhibit traits such as openness, imagination, unconventionality, and curiosity while displaying lower levels of conscientiousness, responsibility, dependability, and order in comparison to shod runners. Additionally, research on middle-aged male runners has identified common personality profiles and associated positive self-perception with long-term involvement in running and training (13). Runners demonstrated heightened intelligence, creativity, self-sufficiency, sobriety, and forthrightness compared to the general population, embodying traits of introversion, shyness, and a propensity for imaginative pursuits in their personality composition (14). Besides, a prospective study found that runners with high scores on the type A behaviour (characterized by agitation, hostility, rapid speech, and an extremely competitive nature) screening questionnaire experienced significantly more injuries, especially multiple injuries (15). Nonetheless, limited information exists on the personality of recreational runners, relying mostly on old studies. This underscores the imperative for contemporary, original research specifically addressing the personality traits of recreational runners (16).*

*Personality traits could be effectively classified into one of two possible categories along various axes using the Myers-Briggs Type Indicator (MBTI) test, a tool rooted in Jungʼs psychology (17). Besides, recent research suggested that the duty factor (DF), i.e., the proportion of time spent in contact with the ground during a running stride, plays a pivotal role in illustrating two distinct spontaneous running forms in recreational runners (18, 19). Low DF runners were shown to exhibit a shorter contact time (t_c_) and larger vertical oscillation of the center of mass during flight time (t_f_), favoring elastic energy reuse. Conversely, high DF runners demonstrated a longer t_c_ and reduced work against gravity to promote forward progression. Both strategies could be efficiently employed at endurance running speeds, leading to similar running economy measures (18).*

*In the present study, we delve into the intriguing realm of embodied cognition by exploring if an intricate connection could exist between personality traits and spontaneous running patterns of shod runners. Indeed, the aim of this study was to explore whether the two categories of personality traits within the various MBTI axes could be linked to biomechanical variables that characterize two distinct running forms naturally embraced by individuals. This exploration should shed light on the complex relationship between who we are and how we run. We hypothesized that personality traits would demonstrate association with spontaneous running patterns.*

Lines 398-408

*Notwithstanding, understanding the connections between personality traits and movement holds potential public health implications. Indeed, tailoring physical interventions through suitable exercises and instructions could mitigate non-adherence (42) and variability in responses (43) to a running training program in the context of a modern sedentary lifestyle. The disparities in running biomechanics associated with sensing and intuition personality traits might result in distinct injury locations or different underlying causes for a given injury. This suggests the need for tailored rehabilitation treatments, as previously advocated (44). These observations partially align with findings from a prospective study, indicating that runners characterized by agitation, hostility, rapid speech, and an extremely competitive nature (Type A behavior) encountered significantly more injuries, particularly multiple injuries (15).*

2. Establish a logical connection between biomechanics and psychology variables rather than introducing measurements of running patterns and the Myers-Briggs Type Indicator.

**Response:** The last two paragraphs of the revised version of the introduction should now establish a better logical connection between biomechanics and psychology variables.

Lines 114-132

*Personality traits could be effectively classified into one of two possible categories along various axes using the Myers-Briggs Type Indicator (MBTI) test, a tool rooted in Jungʼs psychology (17). Besides, recent research suggested that the duty factor (DF), i.e., the proportion of time spent in contact with the ground during a running stride, plays a pivotal role in illustrating two distinct spontaneous running forms in recreational runners (18, 19). Low DF runners were shown to exhibit a shorter contact time (t_c_) and larger vertical oscillation of the center of mass during flight time (t_f_), favoring elastic energy reuse. Conversely, high DF runners demonstrated a longer t_c_ and reduced work against gravity to promote forward progression. Both strategies could be efficiently employed at endurance running speeds, leading to similar running economy measures (18).*

*In the present study, we delve into the intriguing realm of embodied cognition by exploring if an intricate connection could exist between personality traits and spontaneous running patterns of shod runners. Indeed, the aim of this study was to explore whether the two categories of personality traits within the various MBTI axes could be linked to biomechanical variables that characterize two distinct running forms naturally embraced by individuals. This exploration should shed light on the complex relationship between who we are and how we run. We hypothesized that personality traits would demonstrate association with spontaneous running patterns.*

3. Rewrite the sentence on lines 56-58 for better clarity.

**Response:** This sentence was removed in the revised version of the manuscript.

4. Summarize findings from previous studies between different motion and personality traits (lines 63-71).

**Response:** Findings from previous studies between motion and personality traits have been summarized in two paragraphs in the revised version of the introduction.

Lines 79-112

*Exploring the intricate dynamics of embodied cognition sheds light on the reciprocal influence between the mind, body, and the vibrant world they jointly navigate. For example, an investigation revealed that the extraverted-introverted continuum influences upright posture, with 96% of extraverted individuals maintaining an “ideally aligned” posture, while 83% of introverted individuals exhibit a kyphosis-lordosis posture (4). Furthermore, higher levels of extraversion and conscientiousness have been linked to increased physical activity levels (5) and to faster walking speeds (6, 7), suggesting that the way individuals move may reflect their underlying personality traits. Conscientiousness, notably, has shown the capacity to mitigate the age-related decline in walking speed (6, 8). Research has also uncovered that personality traits manifest in an individual’s walking gait (9). For instance, it was observed that greater pelvis motion in the horizontal plane during walking is associated with greater agreeableness in females, while, for males, greater thorax motion in the horizontal plane is linked with extraversion (9). In a recent development, machine learning techniques were employed with notable accuracy to assess personality traits through the analysis of gait recorded using videos (10) or smartphone sensors (11).*

*Extending this line of inquiry into the realm of running, there are indications that personality traits may influence a runner’s likelihood of engaging in barefoot running (12). This study’s results suggest that runners who have attempted barefoot running tend to exhibit traits such as openness, imagination, unconventionality, and curiosity while displaying lower levels of conscientiousness, responsibility, dependability, and order in comparison to shod runners. Additionally, research on middle-aged male runners has identified common personality profiles and associated positive self-perception with long-term involvement in running and training (13). Runners demonstrated heightened intelligence, creativity, self-sufficiency, sobriety, and forthrightness compared to the general population, embodying traits of introversion, shyness, and a propensity for imaginative pursuits in their personality composition (14). Besides, a prospective study found that runners with high scores on the type A behaviour (characterized by agitation, hostility, rapid speech, and an extremely competitive nature) screening questionnaire experienced significantly more injuries, especially multiple injuries (15). Nonetheless, limited information exists on the personality of recreational runners, relying mostly on old studies. This underscores the imperative for contemporary, original research specifically addressing the personality traits of recreational runners (16).*

Methods
1. Clarify if all runners are short-distance runners and provide a clear definition of training hours.

**Response:** Participants were all recreational endurance runners. Moreover, training hours per week was modified to weekly running hours to emphasis the average number of hours participants were running per week. These two remarks were clarified in the revised version of the manuscript.

Lines 136-140

*Eighty recreational endurance runners with regular running training, 67 males (age: 29.3 ± 11.1 years, height: 178.2 ± 6.4 cm, body mass: 72.0 ± 8.5 kg, and weekly running hours: 6.4 ± 3.8 h·week^−1^) and 13 females (age: 29.8 ± 11.6 years, height: 167.2 ± 6.9 cm, body mass: 60.8 ± 9.1 kg, and weekly running hours: 8.5 ± 7.8 h·week^−1^), participated in this study.*

2. Explain why participants were recruited in 2014, and address concerns about the study not being published in the last nine years.

**Response:** We agree with the reviewer that the 9-year period might seem to be a long period of time to publish such work. However, we would like to mention to the reviewer that research is not the full-time job of the authors of the present manuscript because the authors all work in a company. Moreover, the manuscript did several back and forth with reviewers in other journals, which increased the publishing time of this study.

3. Justify the use of 3.3, 4.2, and 5 m/s running speeds (lines 150-151).

**Response:** These three running speeds were chosen to represent low, moderate, and high running speeds.

Lines 158-160

*These running speeds were chosen because they represent low, moderate, and high endurance running speeds.*

4. Provide more details on the lab setting, data reduction, and motion capture methods.

**Response:** We thank the reviewer for this comment. As correctly pointed out by the reviewer, the materials and methods section presenting the lab setting, data reduction, and motion capture methods was lacking some important information. Hence, this part was reviewed in the revised version of the manuscript.

Lines 156-178

*Subsequently, participants completed three randomized 50-m running-trials at speeds of 3.3, 4.2, and 5 m·s^−1^ starting from a standing-still position (2-min rest period between trials). These running speeds were chosen because they represent low, moderate, and high endurance running speeds. Speed was monitored using photoelectric cells (Racetime2, MicroGate, Timing and Sport, Bolzano, Italy) placed at the 20 and 40-m marks. A running trial was accepted if the monitored speed was within ± 5% of the requested speed and repeated otherwise after a 2-min rest period. Less than 15% of the trials were discarded.* *The Optojump^®^ photoelectric cells (MicroGate Timing and Sport, Bolzano, Italy) were used to measure t_c_ (in ms) and t_f_ (in ms) between the 20 and 40-m marks. The cells consist of two parallel bars which were set 1 m apart and were connected to a personal computer. One bar acts as a transmitter unit containing light emitting diodes positioned 3 mm above the ground, whereas the other bar acts as the receiver unit. When the light is interrupted by an individual’s foot during running, a timer within the Optojump system records time with a precision of 1 ms (sampling frequency of 1000 Hz). This allows measuring t_c_ as the time that the light is interrupted and t_f_ as the time between interruptions. As for each participant, the average value over the 20-m distance was computed for t_c_ and t_f_ and used in what follows.*

*Then, swing time (t_s_, in ms), corresponding to the time during which a foot is not in contact with the ground within a running stride, was calculated as two consecutives t_f_ and one t_c_, i.e.,* $t_{s}={2t}_{f}+t_{c}$*.*

Results
1. Address any differences in population characteristics between "Sensing" and "Intuition" groups.

**Response:** Participant characteristics between Sensing and Intuition runners, as suggested by the reviewer, as well as between the other three MBTI axes have been added to the revised version of the manuscript.

*Lines 235-144*

*Classifications of participants along the four MBTI axes are reported in Table 1. Normality and homogeneity of variance were verified for age, height, body mass, and weekly running hours (p ≥ 0.07; Table 1) except for age and weekly running hours which were not normally distributed (p ≤ 0.04; Table 1). ANOVA and non-parametric ANOVA results indicated no main effect of the MBTI axes on age, height, body mass, and weekly running hours (p ≥ 0.07; Table 1), suggesting that these characteristics were similar across each MBTI axis. Effect sizes were small for age, height, body mass, and weekly running hours between categories of the four MBTI axes (|d| ≤ 0.27; Table 1), except for weekly running hours of the extraversion-introversion axis, and height and body mass of the thinking-feeling axis which were moderate (0.40 ≤ |d| ≤ 0.66; Table 1).*

**Table 1.** Participant characteristics (age, height, body mass, and weekly running hours) for the four Myers-Briggs Type Indicator (MBTI) axes together with their Cohen’s *d* effect size. Significant differences (*p* ≤ 0.05) are indicated in bold. Participant characteristics along each MBTI axis were compared using ANOVA and non-parametric ANOVA when data normality was not verified. Data normality and homogeneity of variance were evaluated using Kolmogorov-Smirnov and Levene's tests, respectively.

| MBTI axis | | Group | Number of participants | Age  (y) | Height  (cm) | Body mass  (kg) | Weekly running hours (h/week) |
| --- | --- | --- | --- | --- | --- | --- | --- |
| Extraversion-introversion | | Extraversion | 37 (46%) | 30 ± 12 | 176 ± 8 | 70 ± 9 | 6 ± 4 |
|  |  | Introversion | 43 (54%) | 29 ± 10 | 177 ± 7 | 71 ± 10 | 8 ± 5 |
|  |  |  | *d* | -0.06 | 0.06 | 0.12 | 0.40 |
| Sensing-intuition | | Sensing | 47 (59%) | 30 ± 12 | 177 ± 8 | 70 ± 10 | 6 ± 5 |
|  |  | Intuition | 33 (41%) | 28 ± 10 | 176 ± 7 | 70 ± 9 | 7 ± 5 |
|  |  |  | *d* | -0.15 | -0.08 | -0.02 | 0.11 |
| Thinking-feeling | | Thinking | 35 (44%) | 29 ± 11 | 179 ± 7 | 72 ± 9 | 7 ± 6 |
|  |  | Feeling | 45 (56%) | 29 ± 11 | 174 ± 8 | 68 ± 9 | 6 ± 4 |
|  |  |  | *d* | -0.01 | -0.66 | -0.43 | -0.27 |
| Judging-perceiving | | Judging | 41 (51%) | 29 ± 12 | 177 ± 8 | 70 ± 10 | 7 ± 5 |
|  |  | Perceiving | 39 (49%) | 30 ± 10 | 175 ± 8 | 70 ± 9 | 6 ± 5 |
|  |  |  | *d* | 0.07 | -0.24 | 0.06 | -0.11 |
| *p*-values | ANOVA Main MBTI effect | | | 0.18 | 0.07 | 0.46 | 0.16 |
|  | Normality test | | | **<0.001** | 0.98 | 0.23 | **0.04** |
|  | Homogeneity test | | | 0.31 | 0.82 | 0.82 | 0.74 |

Note. Data are presented as mean ± standard deviation.

2. Double-check values in Table 1, specifically △L.

**Response:** As correctly pointed out by the reviewer, the units of ∆*L* were not corrected and has been modified in the revised version of the manuscript.

3. Report 95% confidence intervals of mean differences in pair-wise comparisons.

**Response:** 95% confidence intervals of mean differences was added for all significant pair-wise comparisons along the MBTI axes, as suggested by the reviewer.

Lines 226-227

*95% confidence intervals [lower, upper] of mean differences (∆s) were calculated for each significant post-hoc comparison along the MBTI axes.*

Lines 272-279

*The sensing-intuition axis reported significant differences among the biomechanical variables, leading to sensing runners showing a longer* $t_{c}$ *(∆ = 13 ms [5 ms, 21 ms]; p = 0.002; small effect size; d = 0.44), shorter* $t_{f}$ *(∆ = -16 ms [-22 ms, -10 ms]; p < 0.001; moderate effect size; d = -0.73), higher DF (∆ = 2.1% [1.3%, 2.9%]; p < 0.001; moderate effect size; d = 0.66), smaller* $k_{leg}$ *(∆ = -1.1 kN [-1.5 kN, -0.7 kN]; p = 0.01; moderate effect size; d = -0.68), and larger* $\Delta L$ *(∆ = 1.4 cm [0.7 cm, 2.1 cm]; p = 0.02; moderate effect size; d = 0.55) than intuition runners (Fig. 1 and Table 1).*

Discussion
1. Include more evidence supporting the findings, considering factors like landing patterns, running cadence, surfaces, and footwear.

**Response:** We thank the reviewer for this comment. Unfortunately, the authors were not able to find any references supporting the findings of the present study, i.e., relation between running biomechanics and personality traits. However, we added references about other biomechanical factors that might reveal differences between different personality traits.

Lines 362-265

*In addition, larger thickness and cross-sectional area of both the Achilles tendon and plantar fascia resulted in lower DF in barefoot running (37). Hence, such investigations might reveal thicker and slenderer lower limbs, as well as shorter heels in intuition than sensing runners.*

Lines 367-370

*Besides, given that DF is associated with foot-strike pattern, the degree of lower limb flexion during stance, and external forces (18, 19, 37, 38), it would be valuable for future studies to investigate the connection between personality traits and these additional biomechanical variables.*

Conclusion
Refine this section for clarity.

**Response:** The conclusion was strengthened based on the main findings of the study.

Lines 441-455

*This exploratory study offers compelling evidence that personality traits, specifically sensing and intuition, are associated with distinct running biomechanics. Sensing runners, who pay attention to physical realities and prefer practical and specific facts, tend to adopt a more grounded running form associated with longer* $t_{c}$*, shorter* $t_{f}$*, higher DF, and larger* $\Delta L$ *than intuition runners. On the contrary, intuition runners, who prefer abstract concepts and theories, and make unconscious connections across their disciplines of knowledge, tend to opt for a more dynamic and springy running form with shorter* $t_{c}$ *and larger* $k_{leg}$ *than sensing runners. These findings implies that runners with sensing and intuition personality traits exhibit variations in their utilization of lower limb structures as springs. Intuition runners appeared to rely more on the stretch-shortening cycle, effectively optimizing their running economy through the reutilization of elastic energy, whereas sensing runners seemed to prioritize forward progression over vertical oscillations to optimize running economy. While this specific study did not permit drawing causal or predictive conclusions, it highlights the fascinating interaction between an individual's personality traits and their preferred movement patterns.*

References
Update recent references.

**Response:** References have been updated according to the suggestion of the reviewer.
