# Supplementary material for: Mind to move: Differences in running biomechanics between sensing and intuition shod runners
Source: PLoS One. 2024 Apr 3;19(4):e0300108. doi: 10.1371/journal.pone.0300108 (PMC10990178; doi:10.1371/journal.pone.0300108)
Supplement: Attachment — Submitted filename: review.docx [file pone.0300108.s003.docx]

**Subject:** Response to the editor and reviewer

**Manuscript title:** Mind to move: differences in running biomechanics between Sensing and Intuition shod runners

We would like to thank the reviewers for the useful comments. The feedback provided was helpful in improving the quality of our manuscript during the revision process. We have adapted our manuscript as suggested based on the reviewers’ comments. We have provided an answer to each comment.

In addition, to facilitate the review process, we have indicated all modifications in the manuscript in RED font color, except for where we removed text. We hope that the reviewers find our responses meet their expectations.

**Reviewer 2**

Despite the extensive revisions made by the author based on previous comments, there are still issues that need to be addressed. I recommend that the author make further modifications based on the following suggestions.

1. The author did not indicate the influence of speed on the results in the abstract.

**Response:** We thank the reviewer for this comment. The influence of running speed on the results was added to the abstract for the post-hoc comparisons between sensing and intuition runners. However, the main effect of running speed on the biomechanical variables was not added to the abstract because this result is not important regarding the aim of the present study.

Lines 55-56

*Post-hoc tests revealed a significant difference in* $t_{c}$ *between intuition and sensing runners at all speeds (p≤0.02).*

2. In terms of previous comment “1) the inclusion criteria were relatively broad, for example, how about the running strike patterns of these participants?”, the running strike patterns would have a significant influence on the lower limb biomechanics of running, such as vertical loading rates of GRF, foot and knee angle, and so on

(<https://www.jospt.org/doi/10.2519/jospt.2015.6019>).

How does the author eliminate this influence? Or has the author considered these effects?

**Response:** We did not consider the foot-strike pattern of participants because the authors believe that the spontaneous running pattern is a global system with several interconnected variables, which therefore does not solely rely on the foot-strike pattern. This statement was added to the materials and methods section of the manuscript. In addition, the influence of the foot-strike pattern on biomechanical variables has been discussed in the limitation section of the manuscript.

Lines 124-128

*Low DF runners were shown to exhibit a shorter contact time (t_c_), larger vertical oscillation of the center of mass during flight time (t_f_), and more anterior (midfoot and forefoot) strike pattern, favoring elastic energy reuse. Conversely, high DF runners demonstrated a longer t_c_ , more rearfoot strike pattern, and reduced work against gravity to promote forward progression.*

Lines 153-156

*However, nothing specific about their spontaneous running pattern such as their foot-strike pattern was required because the running pattern is assumed to be a global system with several interconnected variables (18, 19, 22, 23).*

Lines 454-464

*Finally, this study did not measure the foot-strike pattern of participants, despite existing biomechanical variations reported among different patterns (66, 67). Notably, forefoot and midfoot strikers exhibited significantly shorter contact times* $t_{c}$ *compared to heel strikers (68). However, it's crucial to recognize that the foot-strike pattern is just one element within the broader running pattern, encompassing various interconnected variables (18, 19, 22, 23). Considering this, runners with a more grounded running form, and associated with sensing personality trait, should exhibit a more rearfoot strike pattern because of the longer* $t_{c}$*, while those with a more dynamic and elastic form, often associated with intuition personality trait, should demonstrate a more forefoot/midfoot strike pattern due to the shorter* $t_{c}$*. Nevertheless, this statement requires validation through future research.*

3. “Given that differences in footwear characteristics can underpin differences in running biomechanics (52), using a standardized shoe might have led to different study outcomes in terms of running biomechanics.”, it is suggested that the author cite recent relevant studies to further elaborate on this limitation. I leave to the authors some bibliography to complement this information

(<https://doi.org/10.3390/bioengineering9110607>; <https://doi.org/10.1016/j.jbiomech.2023.111597> ).

**Response:** We thank the reviewers for these references which have been added to the manuscript.

Lines 449-452

*Given that differences in footwear characteristics can underpin differences in running biomechanics* (58-62), *using a standardized shoe might have led to different study outcomes in terms of running biomechanics.*

**Reviewer 4**

**General Comment**

This study aims to contrast the biomechanical variances observed in runners with distinct personality traits, specifically, the sensate type and the intuitional type, during running exercises. The findings illustrated distinct running biomechanics associated with varying personality traits, particularly those related to perception and intuition. The authors' investigation into the intricate relationship between individual personality traits and running patterns is genuinely commendable, yielding substantial results. However, the authors may have overlooked the robustness of the experiment, given its completion in 2014. Additionally, certain aspects of the logical flow in the manuscript seemed somewhat stretched, lacking a plausible explanation, thus falling short of our journal's criteria concerning experimental processes and textual logic. As a result, regrettably, the reviewers deemed this study unsuitable for publication in our journal.

**Response:** We thank the reviewer for this important comment. We agree with the reviewer that our study has some limitations. For this reasons, we have adapted our manuscript as suggested based on the comments of the reviewer. The answers to each comment of the reviewer are reported below.

**Specific comments**

*Abstract*
Minor:
Line50-53: In contrast to individuals with intuition traits, those with sensing traits exhibited significant differences (p≤0.02) in ground contact time, flight time, duty factor, and maximal leg compression. This finding is somewhat surprising.

**Response:** This finding might be surprising but this is actually the main finding of the present study.

Line56-57: Sensing individuals tend to focus on concrete facts and physical realities while intuition individuals emphasize abstract concepts and patterns of information” How did the author measure this particular result?

**Response:** We thank the reviewer for this comment. We would like to mention to the reviewer that this is the definition of sensing and intuition individuals according to MBTI. This has been clarified in the revised version of the manuscript.

Lines 56-59

*According to the definition of each category provided by the MBTI, sensing individuals tend to focus on concrete facts and physical realities while intuition individuals emphasize abstract concepts and patterns of information.*

*1. Introduction*

Major:
The introduction section suggests a motivation for the study based on the scarcity of recent personality surveys on recreational runners, leading the authors to explore the relationship between personality traits and running biomechanics. However, this motivation may not resonate strongly with readers and reviewers. The reviewers recommend that the authors enhance the practical benefits of the study to establish its relevance more effectively.

**Response:** We thank the reviewer for this important comment. The introduction section has been revised to improve the relevance of the present study.

Minor:
Line88-91: For the variations in gait characteristics between males and females, is this due to individual personalities or to gender?

**Response:** We agree with the reviewer that an information was missing in the manuscript to remove the influence of gender. Indeed, there were no significant distinctions in the horizontal motion of the thorax and pelvis between females and males. Hence, these variations in gait characteristics might be influenced by individual personalities, removing the influence of gender.

Lines 92-94

*Since there were no significant distinctions in the horizontal motion of the thorax and pelvis between females and males, these correlations might be influenced by individual personalities, thus removing the influence of gender (9).*

Line101-103: The authors were advised to incorporate more recent studies in the field, preferably from the last 5 years, to better capture the contemporary relationship between an individual's personality and their choice of sport. Utilizing more recent literature would enhance the relevance and applicability of the findings to current circumstances.

**Response:** We thank the reviewer for this comment. We agree with the reviewer that more recent studies in the field should be incorporated to the manuscript. However, to the best of the authors’ knowledge, there is no such study. This has been further acknowledged in the revised version of the manuscript.

Lines 111-114

*Nonetheless, limited information on the personality of recreational runners is available, primarily derived from older studies, and to the best of the author’s knowledge, with no recent research on this topic.*

Line114-116: Why did the authors choose the MBTI test instead of the Big Five Inventory? As far as the reviewers realized, the reliability and validity of the MBTI has many psychometric limitations and is therefore not recommended for widespread academic use.

Boyle G J. Myers‐Briggs type indicator (MBTI): some psychometric limitations. Australian Psychologist, 1995, 30(1): 71-74.

Stein R, Swan A B. Evaluating the validity of Myers‐Briggs Type Indicator theory: A teaching tool and window into intuitive psychology. Social and Personality Psychology Compass, 2019, 13(2): e12434.

**Response:** The authors opted for the MBTI over the Big Five Inventory due to the nuanced nature of the MBTI's axes, which assign two distinct categories, as opposed to the Big Five, which merely indicates the presence of a trait. As researchers, we assert that the Big Five tends to involve value judgments, whereas the MBTI attributes one of two potential personality traits for each axis. However, we agree with the reviewer that the MBTI validity has been questioned. Nonetheless, MBTI correlates well with the big five. This was added to the limitation paragraph in the discussion section of the manuscript.

Lines 421-433

*Besides, the MBTI validity has been questioned (49) and is regarded as a controversial approach (50), with psychometric limitations (51, 52). Nevertheless, this tool is still the most widely used personality assessment in the world (30, 36). Moreover, MBTI correlates well with the Neuroticism, Extraversion, Openness (NEO) Personality Inventory, another widely used personality assessment tool that examines the Big Five personality traits (53, 54) and MBTI has been utilized, though several decades ago, to assess personality traits in middle-age male runners (13). The MBTI was preferred over the Big Five in the present study due to its nuanced nature. The MBTI assigns a personality trait among two distinct categories for each axis, as opposed to the Big Five, which merely indicates the absence or presence of a given personality trait. As researchers, we assert that the Big Five tends to involve value judgments, whereas the MBTI assigns one of two possible personality traits to each axis without implying superiority for either.*

Line121-123: Generally, there is a possibility that the extended contact time observed in athletes could be a strategy employed to conserve energy during running.

Lempke A F D J, Hunt D L, Willwerth S B, et al. Biomechanical changes identified during a marathon race among high-school aged runners. Gait & Posture, 2024, 108: 44-49.

**Response:** As correctly pointed out by the reviewer, decreasing and increasing ground contact time could be two opposite but efficient strategies to improve running economy. This statement was added to the revised version of the manuscript.

Lines 128-132

*Similarly, decreasing and increasing t_c_ could represent two opposing yet efficient strategies for enhancing running economy (20, 21). The first strategy involves an increase in vertical stiffness to improve running economic (20) while the second strategy posits that generating force over a longer period might be more economical (21).*

As the author discusses commonly employed running skills among athletes, it remains unclear how the author correlates these skills with athlete personality traits.

**Response:** We thank the reviewer for this comment. We agree with the reviewer that it was unclear how running biomechanics was correlated with athletes personality traits. Hence, we added to the revised version of the manuscript that there is no superior category in each MBTI axis. Additionally, as there are two spontaneous running forms that could be efficiently employed at endurance running speeds, this allowed us making an hypothetical link between MBTI category and spontaneous running form. This was also added to the revised manuscript for clarification.

Line 119

*Notably, there is no superior category in each MBTI axis.*

Lines 132-134

*Consequently, one may contemplate whether each of these running strategies could be associated with a specific personality trait category.*

Line125-127: The reviewer suggests that the author consider slightly restructuring the statement to make it more fluent: In the present study, we delve into the intriguing realm of embodied cognition by exploring whether an intricate connection could exist between personality traits and the spontaneous running patterns of shod runners.

**Response:** We thank the reviewer for this suggestion, which has been added to the revised version of the manuscript.

Lines 136-138

*In the present study, we delve into the intriguing realm of embodied cognition by exploring whether an intricate connection could exist between personality traits and the spontaneous running patterns of shod runners.*

*2. Materials and Methods*

Minor:
Line140: What is the author's purpose in mentioning "white" Caucasians here?

**Response:** Please note that we removed the term “white” in the corresponding sentence.

Line140-142: In the description of the participants above, the average amount of time the subjects ran per week was approximately 6 hours for males and 8 hours for females. Here the authors only asked the participants to perform at least 1 hour per week, does the intensity of the training meet the training requirements of the participants? What is the authors' basis for setting 1 hour?

**Response:** The researchers aimed to enlist recreational runners not necessarily engaging in more than one hour of running per week. Surprisingly, participants recruited in the present experiment demonstrated a consistent weekly running duration of approximately 6 and 8 hours for male and female, respectively. Despite the experimental protocol requiring a 50 m run at 18 km/h, the authors were confident that the runners' training regimens adequately prepared them to successfully carry it out, especially because there was no physiological restriction during the measurements.

Line142-143: Why were the authors not specific in terms of participants' foot impact patterns? As mentioned in previous studies, there may be a greater correlation between foot impact pattern, running economy, and ground contact time

Hayes P, Caplan N. Foot strike patterns and ground contact times during high-calibre middle-distance races. Journal of sports sciences, 2012, 30(12): 1275-1283;

Di Michele R, Merni F. The concurrent effects of strike pattern and ground-contact time on running economy. Journal of science and medicine in sport, 2014, 17(4): 414-418;

Almeida M O, Davis I S, Lopes A D. Biomechanical differences of foot-strike patterns during running: a systematic review with meta-analysis. Journal of Orthopaedic & Sports Physical Therapy, 2015, 45(10): 738-755.

Therefore, the reviewers recommended that the authors should screen participants on their foot strike patterns.

**Response:** We did not consider the foot-strike pattern of participants because the authors believe that the spontaneous running pattern is a global system with several interconnected variables, which therefore does not solely rely on the foot-strike pattern. This statement was added to the materials and methods section of the manuscript. In addition, the influence of the foot-strike pattern on biomechanical variables has been discussed in the limitation section of the manuscript.

Lines 124-128

*Low DF runners were shown to exhibit a shorter contact time (t_c_), larger vertical oscillation of the center of mass during flight time (t_f_), and more anterior (midfoot and forefoot) strike pattern, favoring elastic energy reuse. Conversely, high DF runners demonstrated a longer t_c_ , more rearfoot strike pattern, and reduced work against gravity to promote forward progression.*

Lines 153-156

*However, nothing specific about their spontaneous running pattern such as their foot-strike pattern was required because the running pattern is assumed to be a global system with several interconnected variables (18, 19, 22, 23).*

Lines 454-464

*Finally, this study did not measure the foot-strike pattern of participants, despite existing biomechanical variations reported among different patterns (66, 67). Notably, forefoot and midfoot strikers exhibited significantly shorter contact times* $t_{c}$ *compared to heel strikers (68). However, it's crucial to recognize that the foot-strike pattern is just one element within the broader running pattern, encompassing various interconnected variables (18, 19, 22, 23). Considering this, runners with a more grounded running form, and associated with sensing personality trait, should exhibit a more rearfoot strike pattern because of the longer* $t_{c}$*, while those with a more dynamic and elastic form, often associated with intuition personality trait, should demonstrate a more forefoot/midfoot strike pattern due to the shorter* $t_{c}$*. Nevertheless, this statement requires validation through future research.*

Line 143-145: As a human experiment, all participants were entitled to be informed about the content of the experiment in which they were participating.

**Response:** We thank the reviewer for this comment. Participants knew the content of the two experimental sessions as they provided written informed consent to perform a running biomechanical analysis and a personality traits assessment. Nonetheless, they were not informed that the purpose was to investigate the relationship between personality traits and biomechanics. Hence, this specific part was removed from the sentence in the revised version of the manuscript.

Line 147-149: As the authors acknowledged, this experiment was conducted in 2014. Given the recent surge in long-distance running activities, such as marathons and trail running, involving individuals with diverse personalities, what contemporary relevance and application can be attributed to the authors' research a decade later?

**Response:** We would like to mention to the reviewer that the surge in long-distance running activities was already present a decade ago. For instance, the following blog <https://ade3.medium.com/bostons-evolution-1897-2018-cdd91aa79f95> depicts a plateau in the number of participants (men and women) at the Boston marathon in the last few years. Moreover, the same blog shows a plateau in the finishing time of men and women.

Furthermore, as runners are represented by individuals with diverse personalities, it could be of interest to explore if personality traits are related to running biomechanics. Such relations could have public health implications. Indeed, tailoring physical interventions through suitable exercises and instructions could mitigate non-adherence and variability in responses to a running training program in the context of a modern sedentary lifestyle. This statement was already part of the discussion section.

Lines 404-411

*Notwithstanding, understanding the connections between personality traits and movement holds potential public health implications. Indeed, tailoring physical interventions through suitable exercises and instructions could mitigate non-adherence (45) and variability in responses (46) to a running training program in the context of a modern sedentary lifestyle. The disparities in running biomechanics associated with sensing and intuition personality traits might result in distinct injury locations or different underlying causes for a given injury. This suggests the need for tailored rehabilitation treatments, as previously advocated (47).*

Line151-152: Why did the authors not make any special requirements regarding the participants' running shoes? Previous studies have shown that different running shoes, especially for longer distances, provide an essential role in running economy and individualized performance.

Roca-Dols A, Losa-Iglesias M E, Sánchez-Gómez R, et al. Effect of the cushioning running shoes in ground contact time of phases of gait. Journal of the mechanical behavior of biomedical materials, 2018, 88: 196-200;

De Wit B, De Clercq D, Lenoir M. The effect of varying midsole hardness on impact forces and foot motion during foot contact in running. Journal of applied biomechanics, 1995, 11(4): 395-406.

Hence, if participants had utilized distinct running shoes, it would have evidently introduced additional variables during the experiment.

**Response:** We agree with the reviewer that using a standardized shoe might have led to different study outcomes in terms of running biomechanics. However, recreational runners are more comfortable wearing their own shoes, and show individual responses to novel footwear and cushioning properties. Nevertheless, this is discussed in the limitation section of the manuscript, where the references provided by the reviewer have also been added for completeness.

Lines 449-452

*Given that differences in footwear characteristics can underpin differences in running biomechanics (58-62), using a standardized shoe might have led to different study outcomes in terms of running biomechanics.*

Line158-160: What is the author's criteria for judging these three speeds as "low, medium, and fast"?

**Response:** We agree with the reviewer that this statement was vague. Therefore, we modified this sentence to mention that the three running speeds employed represent the 10-km race pace of most of endurance runners.

Lines 170-172

*These running speeds were chosen because they represent the 10-km race pace of most of endurance runners (24).*

Line 186-187: What is the basis for the authors' use of this formula for calculating the participant's maximum vertical ground reaction force?

**Response:** The basis of this formula is the sine-wave model for the vertical ground reaction. This was developed by Morin et al. and was further emphasized in the manuscript.

Lines 196-201

*Furthermore, the spring-mass characteristics of the lower limb were estimated using a sine-wave model as defined by Morin, Dalleau (28). More explicitly, leg stiffness (k_leg_) was calculated as:*

$$k_{leg}=\frac{F_{max}}{\Delta L}.$$

$F_{max}$ *represents the maximal vertical ground reaction force and was estimated using* $F_{max}=mg\frac{\pi}{2}\left( \frac{t_{f}}{t_{c}}+1 \right)$*, where m is body mass and g is gravitational acceleration (28).*

Line190: The article lacks clarification on the model and device used by the authors to capture the subject's center of mass.

**Response:** There was no device to capture the center of mass. All biomechanical variables reported in the present study are estimated from ground contact time and flight time. This was clarified in the revised version of the manuscript.

Lines 188-195

*t_c_ and t_f_ were the basis of the other biomechanical variables computed herein. SF was calculated as:*

$$SF=\frac{1}{t_{c} + t_{f}},$$

*and DF, reflecting the relative contribution of t_c_ during the running stride, as (26, 27):*

$$DF=\frac{t_{c}}{(t_{c}+t_{s})},$$

*where t_s_ represents the swing time, which is the time during which a foot is not in contact with the ground within a running stride, and is calculated as two consecutives t_f_ and one t_c_, i.e.,* $t_{s}={2t}_{f}+t_{c}$*.*

Lines 204-206

*where* $\Delta z$ *is the absolute displacement of the center of mass during stance estimated using* $\Delta z=\frac{F_{max}}{m}\frac{t_{c}^{2}}{\pi^{2}}-g\frac{t_{c}^{2}}{8}$ *(28),* $s$ *the running speed, and* $L$ *the participant’s leg length, estimated as 0.53 of body height (28, 29).*

Line191-192: What is the basis for the 0.53 value? Please cite relevant literature and adapt it to this study.

**Response:** References for the 0.53 coefficient have been added to the revised version of the manuscript. We would like to mention to the reviewer that we followed the sine-wave model developed by Morin et al. in the present study.

Lines 204-206

*where* $\Delta z$ *is the absolute displacement of the center of mass during stance estimated using* $\Delta z=\frac{F_{max}}{m}\frac{t_{c}^{2}}{\pi^{2}}-g\frac{t_{c}^{2}}{8}$ *(28),* $s$ *the running speed, and* $L$ *the participant’s leg length, estimated as 0.53 of body height (28, 29).*

Line206-207: The author is kindly asked to add an explanation for the first occurrence of the abbreviation (NEO)

**Response:** This sentence was moved to the limitation section of the revised version of the manuscript and the definition of the NEO acronym was also added.

Lines 424-428

*Moreover, MBTI correlates well with the Neuroticism, Extraversion, Openness (NEO) Personality Inventory, another widely used personality assessment tool that examines the Big Five personality traits (53, 54) and MBTI has been utilized, though several decades ago, to assess personality traits in middle-age male runners (13).*

Line209-210: The study on runners referenced by the authors was conducted in 1991, a notably different era influenced by changes in running technology and societal shifts. Additionally, it remains unclear if there is a correlation between enhancing team cohesion, understanding team dynamics, and the findings of this study. Line210-213: Can the authors elaborate on the connection between the personalities of these students and the recreational runners recruited for this study?

**Response:** We thank the reviewer for this comment. As correctly pointed out by the reviewer, these references did not make sense. Hence, they were removed from the manuscript.

Line216-218: The authors were asked to add tools for sample calculations.

**Response:** The authors added the tool that was used to perform sample size calculations in the revised version of the manuscript.

Lines 222-226

*Sample size calculations determined that 80 participants were required for this study, assuming moderate effect sizes (~0.5) for biomechanical differences between MBTI axes, an α error of 0.05, and a power of 0.8 (31) and was obtained using G*Power (v3.1, available at* [*https://www.psychologie.hhu.de/arbeitsgruppen/allgemeine-psychologie-und-arbeitspsychologie/gpower*](https://www.psychologie.hhu.de/arbeitsgruppen/allgemeine-psychologie-und-arbeitspsychologie/gpower)*) (32)*

*3. Results*

Minor:
The authors are requested to enhance the clarity of Figure 1 and 2, furthermore, the authors do not seem to have mentioned in the methods how the standardization of the phases will be done, which the authors should be kindly requested to add.

**Response:** Figure 1 has been enhanced to improve its clarity while Figure 2 has been removed in the revised version of the manuscript. Figure 2was removed because it did not bring anything relevant. Hence, the standardization of the phases did not need to be explained in the methods section.

Line288: In reference to ∆ in Table 3, the findings of the authors indicate that the most significant alteration in ∆ transpired at higher velocities. In practical training scenarios, ∆ appears to exhibit a declining tendency with escalating running speeds.

Jaén-Carrillo D, Roche-Seruendo L E, Felton L, et al. Stiffness in running: a narrative integrative review. Strength & Conditioning Journal, 2021, 43(2): 104-115.

**Response:** We would like to kindly mention to the reviewer that the increase in ∆*L* with increasing running speed does make sense. Indeed, according to the reference provided by the reviewer, *k*_leg_ should remain constant with increasing running speed, which is also what was observed in the present study. Hence, as *k*_leg_ is the ratio between the peak vertical ground reaction force and ∆*L* and as the peak vertical ground reaction force increases with increasing running speed, ∆*L* should also increase with running speed to keep this ratio constant.

*4. Discussion*

Minor:
Line324-327: What does the author base this conclusion on?

**Response:** This conclusion was actually the definition provided by the MBTI for sensing individuals. This was emphasized in the revised version of the manuscript.

Lines 335-338

*These individuals, according to the definition provided by the MBTI, should pay attention to physical realities and prefer practical and specific facts, preferably something they could perceive with their physical senses (17, 36). Hence, individuals with a more grounded running form should focus on practical facts (sensing individuals).*

Line330-334: The author is kindly asked to redescribe the sentence to make it clearer, as well as to correct its tenses.

**Response:** The sentence was rewritten according to the suggestion of the reviewer.

Lines 343-347

*The more elastic running form of intuition than sensing runners, along with their larger* $k_{leg}$*, suggested that the re-use of elastic energy was an inherent feature of intuition runners. These individuals were better able to use their lower limb structures as springs, representing one of the multiple functional roles of the musculoskeletal system (37).*

Line341-342: What indicators are the authors basing their observations on?

**Response:** This observation was actually the definition provided by the MBTI for intuition individuals. This was emphasized in the revised version of the manuscript.

Lines 354-358

*These individuals, according to the definition provided by the MBTI, should pay attention to the meaning and patterns of information, prefer abstract concepts and theories, and make unconscious connections across their disciplines of knowledge (17, 36). Hence, individuals with a more dynamic and elastic running form should focus on abstract things (intuition individuals).*

Line344: Why does Figure 2 appear here?

**Response:** As correctly pointed out by the reviewer, there was no reason to have the Figure 2 in the discussion section of the manuscript. Moreover, as this figure did not add anything relevant to the present manuscript, it has been removed from the manuscript.

*5. Conclusions*

Major
Reviewers anticipate authors to succinctly summarize and expound upon the tangible outcomes of this study, eschewing vague remarks

**Response:** We thank the reviewer for this comment. The conclusions were shorten to only succinctly summarize the outcomes of the present study.

Lines 467-474

*This exploratory study offers compelling evidence that personality traits, specifically sensing and intuition, are associated with distinct running biomechanics. Sensing runners, who pay attention to physical realities and prefer practical and specific facts, tend to adopt a more grounded running form associated with longer* $t_{c}$*, shorter* $t_{f}$*, higher DF, and larger* $\Delta L$ *than intuition runners. On the contrary, intuition runners, who prefer abstract concepts and theories, and make unconscious connections across their disciplines of knowledge, tend to opt for a more dynamic and springy running form with shorter* $t_{c}$ *and larger* $k_{leg}$ *than sensing runners.*
