# Supplementary material for: Mind to move: Differences in running biomechanics between sensing and intuition shod runners
Source: PLoS One. 2024 Apr 3;19(4):e0300108. doi: 10.1371/journal.pone.0300108 (PMC10990178; doi:10.1371/journal.pone.0300108)
Supplement: Attachment — Submitted filename: review.docx [file pone.0300108.s004.docx]

**Subject:** Response to the editor and reviewer

**Manuscript title:** Mind to move: differences in running biomechanics between Sensing and Intuition shod runners

We would like to thank the reviewers for the useful comments. The feedback provided was helpful in improving the quality of our manuscript during the revision process. We have adapted our manuscript as suggested based on the reviewers’ comments. We have provided an answer to each comment.

In addition, to facilitate the review process, we have indicated all modifications in the manuscript in RED font color, except for where we removed text. We hope that the reviewers find our responses meet their expectations.

**Reviewer 4**

**General Comment**

The reviewers express delight in witnessing the meticulous adaptation and revision of the article's proposal by the authors, leading to enhanced logical coherence and methodological rigor. This research article is commended for its innovative propositions, and the reviewer encourages the author to strive for the utmost professionalism and perfection in presenting their work.

**Response:** We thank the reviewer for this very positive general feedback.

**Specific comments**

*Abstract*

Major: Authors are kindly requested to furnish the essential abbreviations in the abstract section, with a particular emphasis on the depiction of methods, results, and conclusions.

**Response:** As suggested by the reviewer, we have added the abbreviations representing the biomechanical variables used in the present manuscript in the abstract.

Lines 43-47

*To do so, 80 runners completed three randomized 50-m running-trials at 3.3, 4.2, and 5m/s during which their running biomechanics [ground contact time (*$t_{c}$*), flight time (*$t_{f}$*), duty factor (DF), step frequency (SF), leg stiffness (k_leg_), maximal vertical ground reaction force (*$F_{max}$*), and maximal leg compression of the spring during stance (*$\Delta L$*)] was evaluated.*

Lines 52-56

*Individuals classified as sensing demonstrated a more grounded running style characterized by prolonged* $t_{c}$*, shorter* $t_{f}$*, higher DF, and greater* $\Delta L$ *compared to intuition individuals (p≤0.02). Conversely, intuition runners exhibited a more dynamic and elastic running style with a shorter* $t_{c}$ *and higher k_leg_ than their sensing counterparts (p≤0.02).*

*Introduction*

Minor:

- Lines 100-103: The reviewer raised concerns regarding the authors' mention of an association between population characteristics and shoeing habits. As per the methodology described in the article, all subjects had their own footwear, but no survey of footwear was conducted. Therefore, the description of the context of the study lacks real relevance to the study itself, and the reviewer recommends its deletion.

**Response:** We thank the reviewer for this comment. As suggested by the reviewer, this section of the manuscript has been removed.

- Lines 124-128: In this section, the authors discuss the effect of heel strike patterns on running performance. Obviously, this contradicts the authors' response that the spontaneous running pattern is a whole system with multiple interrelated variables and is therefore not entirely dependent on the walking pattern.

**Response:** We would like to mention to the reviewer that we do not discuss the effect of heel strike patterns in this section. However, we describe the biomechanical characteristics of low and high duty factor (DF) runners. DF could be considered as a global variable to describe the running pattern. We further mention that high DF runners have a more rearfoot strike pattern while low DF runners have a more anterior (midfoot and forefoot) strike pattern. This means that the foot-strike pattern is one variable among several that is encapsulated within the categorization made using the DF. Nonetheless, we slightly modified the manuscript to emphasize what has just been written.

Lines 116-123

*Additionally, recent research suggested that the duty factor (DF) plays a pivotal role in illustrating two distinct spontaneous running forms in recreational runners, i.e., runners with either low or high DF (17, 18). DF represents the proportion of time spent in contact with the ground during a running stride and could be considered as a global variable to describe the running pattern. Both running forms (low or high DF runners) could be efficiently employed at endurance running speeds, leading to similar running economy measures (17).*

*Patients and Methods*

Minor:

- Lines 151-153: In the author's response, the author objectively argues that a training regimen of at least one hour per week is sufficient for successful completion of the program. The reviewer would like to emphasize that such a training requirement is clearly too low to meet the normal training needs of the subjects. Additionally, the concept seems relatively abstract, and it is possible that some subjects completed more than 8 hours of training despite the experiment's demands. The reviewers would have appreciated the authors specifying the exact training intensity of the subjects during the week prior to the experiment.

**Response:** We thank the reviewer for this comment. We would like to mention to the reviewer that we sought a heterogeneous panel of runners with varying training backgrounds. Moreover, the authors did not think there could be a problem for the 50-m running trial at 18 km/h, especially because there was no physiological restriction during the measurements. To confirm this, no participants showed difficulty in running at the requested paces. These statements have been added to the manuscript.

Lines 149-153

*To ensure diverse participation in the study, we sought a heterogeneous panel of runners with varying training backgrounds. Consequently, participants were only mandated to run a minimum of one hour per week and maintain good self-reported general health, without any current or recent (<6 months) musculoskeletal injuries.*

Lines 173-174

*No participants showed difficulty in running at the requested paces.*
